# Both Low and High Postprocedural hsCRP Associate with Increased Risk of Death in Acute Coronary Syndrome Patients Treated by Percutaneous Coronary Intervention

**DOI:** 10.1155/2020/9343475

**Published:** 2020-04-17

**Authors:** Runzhen Chen, Chen Liu, Peng Zhou, Yu Tan, Zhaoxue Sheng, Jiannan Li, Jinying Zhou, Yi Chen, Li Song, Hanjun Zhao, Hongbing Yan

**Affiliations:** ^1^Fuwai Hospital, National Center for Cardiovascular Diseases, Peking Union Medical College and Chinese Academy of Medical Sciences, Beijing, China; ^2^Fuwai Hospital, Chinese Academy of Medical Sciences, Shenzhen, China

## Abstract

**Background:**

Inflammation poses dual effects after myocardial infarction, but robust evidence shows that high-sensitivity C-reactive protein (hsCRP), as an inflammatory marker, is constantly associated with worse outcomes. This study is aimed at investigating the probable nonlinear association between postprocedural hsCRP and mortality in patients with acute coronary syndromes (ACS) treated by percutaneous coronary intervention (PCI).

**Methods:**

A total of 3940 consecutive ACS patients treated by PCI with postprocedural hsCRP measurements were retrospectively recruited. Patients were stratified into 5 groups according to quintiles of hsCRP. Cox regression with adjustments for multiple covariates was used for outcome analysis. Restricted cubic spline (RCS) analysis was used to allow possible nonlinear associations. The primary outcome was all-cause death.

**Results:**

During a median follow-up of 727 days, mortality occurred in 207 (5.3%) patients. Adjusted hazard ratio (HR) was higher in the lowest (<2.26 mg/L, HR: 1.90, 95% confidence interval (CI): 1.08-3.33; *P* = 0.025), second highest (10.16-12.56 mg/L, HR: 1.86, 95% CI: 1.09-3.16; *P* = 0.023), and highest quintiles (≥12.56 mg/L, HR: 2.02, 95% CI: 1.21-3.36; *P* = 0.007) of postprocedural hsCRP, compared to the second lowest quintile (2.26-4.85 mg/L). RCS analysis depicted a J-shaped association between postprocedural hsCRP and mortality (*P* for nonlinearity = 0.004). Similar association was observed between hsCRP and cardiac death (*P* for nonlinearity = 0.014), but not for noncardiac mortality (*P* for nonlinearity = 0.228).

**Conclusions:**

Both low and high postprocedural hsCRP were associated with higher risk of death in ACS patients treated by PCI.

## 1. Introduction

High-sensitivity C-reactive protein (hsCRP), a sensitive serum marker of acute and chronic systemic inflammation [1, 2], is closely associated with outcomes of patients with various types of coronary heart diseases (CHD), including stable CHD and myocardial infarction (MI), and those undergoing percutaneous coronary intervention (PCI) [3–6]. Therefore, inflammation was generally considered detrimental in the context of atherosclerosis. However, accumulating evidence from animal and clinical studies shows that complete suppression of inflammation after ischemic myocardial damage could hamper the myocardial healing and even drive up mortality [7–9]. Therefore, a nonlinear relation, instead of a simple linear one, could exist between the intensity of inflammation and risk of adverse events. Due to the delay in hepatic synthesis, perioperative measurements of hsCRP might not fully reflect the inflammation triggered by myocardial necrosis [2, 3]. Besides, the widely used PCI procedures could cause further myocardial damage and result in significant elevation of hsCRP [10, 11]. In this scenario, postprocedural hsCRP might be more appropriate for evaluating the inflammatory response caused by acute coronary events and relevant treatments. However, few studies have evaluated the prognostic value of postprocedural hsCRP after PCI in patients with acute coronary syndromes (ACS), which makes therapeutic windows for anti-inflammatory therapies remain unclear [6, 12]. Therefore, the aim of the current study was to determine the association between postprocedural hsCRP and outcomes of ACS patients treated by PCI.

## 2. Methods

### 2.1. Study Design and Population

This retrospective study was based on a prospective cohort in a large-volume PCI center at a national tertiary care institute specializing in cardiovascular diseases (Fuwai Hospital, Beijing, China). The cohort was maintained by the catheterization laboratory, which has enrolled all patients undergoing coronary angiography and PCI procedures. All the ACS patients undergoing emergent coronary angiography and PCI were included in the original cohort. The patients with ACS consisted of ST-segment elevation MI (STEMI) and non-ST-elevation ACS (NSTE-ACS). Diagnosis and classification of ACS were made according to guidelines and universal definitions up to date, including criteria of clinical presentation, typical characteristics on electrocardiography, dynamical changes of cardiac enzymes, and image evidence [13–15]. Patients with acute infection or inflammatory disorders, missing hsCRP tests results, or unavailable follow-up records were excluded. The study was performed in accordance with the Declaration of Helsinki and was approved by the Ethics Committee of the institute. All subjects provided written informed consent during hospitalization regarding the use of clinical data for the purpose of scientific research by the institute.

From January 2010 to June 2017, the institute had admitted 4760 ACS patients who underwent emergent coronary angiography. Among these patients, 609 did not receive PCI, 37 were complicated with acute infection or inflammatory disorders, 69 had missing postprocedural hsCRP test results, while another 105 had no records of phone call interview, outpatient visits, or rehospitalization at the institute. Finally, a total of 3940 ACS patients treated by PCI were included into the current analysis.

### 2.2. Laboratory Measurements

After the PCI procedure for ACS, blood samples for hsCRP tests were routinely collected via the cubital vein at 6 a.m. on the next morning after patients were admitted into the coronary care unit, in order to allow an overnight fasting state. The level of hsCRP was measured using immunoturbidimetry (Beckmann Assay, Bera, California).

### 2.3. Outcomes and Follow-Up

The primary outcome for the analysis was all-cause mortality. The secondary outcomes included cardiac death and noncardiac death. Outcome data was collected through the routine follow-up, either by outpatient visits or phone call. Patients have been routinely followed up at 1, 6, and 12 months after the discharge. For those who survived more than a year, the subsequent follow-up would be made annually.

### 2.4. Statistical Analysis

Univariable Cox regression model was used to assess the association between levels of hsCRP and mortality, followed by multivariable adjustments. To allow possible nonlinear association, restricted cubic spline (RCS) was used in the analysis for relation between hazard and hsCRP as continuous variables. The models were adjusted for variables that showed a trend for significant difference (*P* < 0.1) in univariable analysis, including age, sex, hypertension, diabetes mellitus, heart failure (Killip classes II-IV), ST-segment elevation, history of PCI, low-density lipoprotein cholesterol (LDL-C), creatinine, pre-PCI Thrombolysis in Myocardial Infarction (TIMI) grade flow of 0, door-to-balloon (D2B) time, culprit lesion, thrombus aspiration, use of intra-aortic balloon pump (IABP), and use of glycoprotein IIb/IIIa (GP IIb/IIIa) inhibitors and aspirin. Categorical variables are presented as numbers (%) and analyzed with chi-square tests. Continuous variables are presented using mean ± SD if they follow the normal distribution and tested with one-way analysis of variance. Otherwise, they are presented as medians with 25^th^ and 75^th^ percentiles and tested by nonparametric Kruskal-Wallis rank sum tests. Statistical analyses were performed using Stata 15.0 (StataCorp, College Station, TX, USA). A *P* value < 0.05 was considered statistically significant.

## 3. Results

### 3.1. Patient Cohort and Baseline Characteristics

Among the 3940 ACS patients treated by PCI, the mean age was 59.0 ± 11.9 years old, and 3105 (78.8%) patients were male (Table 1). Overall, the postprocedural hsCRP was 7.24 (2.77-12.06) mg/L, with an interval of 16.08 (9.38-21.45) hours between admission and blood sample collection. Patients in higher quintiles of postprocedural hsCRP tended to be older and female, while presenting more severe signs of congestive heart failure and higher level of creatinine. They were also more likely to present ST-segment elevation and TIMI 0 flow before PCI, but less likely for a history of previous PCI. D2B time was longer in higher quintiles, while the use of thrombus aspiration, IABP, and GP IIb/IIIa inhibitors was more frequent during the PCI procedure. The presence of hypertension was also different across quintiles. Although patients with NSTE-ACS presented clinical and angiographic profiles of greater risk (Table 2), the hsCRP level of STEMI patients was significantly higher than that of patients with NSTE-ACS (7.55 (2.82-12.16) mg/L vs. 5.55 (2.52-11.30) mg/L, *P* = 0.002). During a median follow-up of 727 days, there were 207 deaths (5.3%), among which 136 cases were cardiac deaths (3.5%) and the other 71 (1.8%) cases were noncardiac deaths.

### 3.2. Nonlinear Association between Postprocedural hsCRP and Outcomes

**Figure 1:**
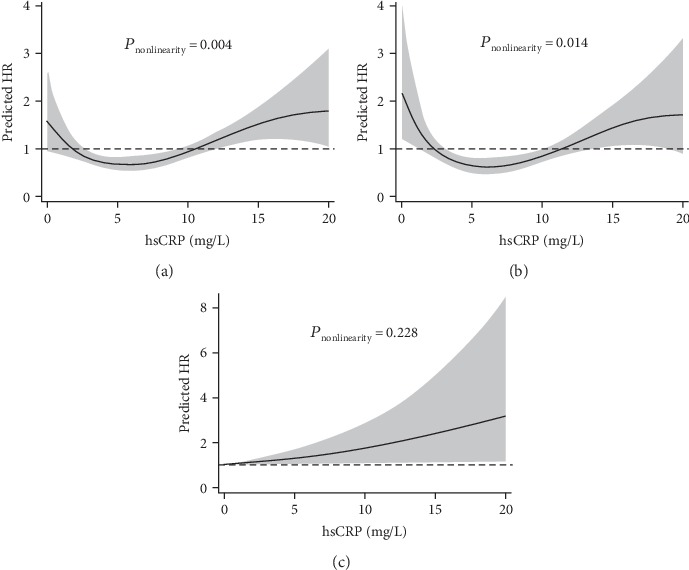
Continuous hazard ratio across hsCRP for all-cause death (a), cardiac death (b), and noncardiac death (c). HR: hazard ratio; hsCRP: high-sensitivity C-reactive protein.

Patients were stratified into 5 groups according to the quintiles of postprocedural hsCRP (i.e., 2.26, 4.85, 10.16, and 12.56 mg/L). The crude incidence rate of death was lowest in the second quintile (2.26-4.85 mg/L) of hsCRP (Table 3). Unadjusted Cox analysis showed a nonlinear relationship between postprocedural hsCRP and mortality, which was significant after multiple adjustments. Patients in the first (<2.26 mg/L, hazard ratio (HR): 1.90, 95% confidence interval (CI): 1.08-3.33; *P* = 0.025), fourth (10.16-12.56 mg/L, HR: 1.86, 95% CI: 1.09-3.16; *P* = 0.023), and fifth (≥12.56 mg/L, HR: 2.02, 95% CI: 1.21-3.36; *P* = 0.007) quintiles gained significantly higher risk of death compared to the second one (2.26-4.85 mg/L), while mortality of patients in the third quintile did not show significant difference (4.85-10.16 mg/L, HR: 1.31, 95% CI: 0.75-2.30; *P* = 0.337). For cardiac death, patients in the lowest quintile even presented higher HR (<2.26 mg/L, HR: 2.39, 95% CI: 1.19-4.80; *P* = 0.014) compared to the highest quintile (≥12.56 mg/L, HR: 1.96, 95% CI: 1.01-3.77; *P* = 0.045). For the subgroup analysis, although the nonlinear association was more significant in STEMI patients (Table 4), no interactions were found between ACS classifications and hsCRP quintiles on all-cause (*P* for interaction = 0.708) or cardiac mortality (*P* for interaction = 0.509). RCS analysis depicted a J-shaped relation between hsCRP and HR for all-cause (*P* for nonlinearity = 0.004) and cardiac death (*P* for nonlinearity = 0.014) (Figure 1). The nadir hsCRP for lowest risk was 5.5 mg/L and 6.0 mg/L for all-cause death and cardiac mortality, respectively. Although higher postprocedural hsCRP was associated with noncardiac death, it was not an independent risk factor after multiple adjustments. RCS analysis did not support a nonlinear association between hsCRP and noncardiac death (*P* for nonlinearity = 0.228).

## 4. Discussions

The major finding of this study was the nonlinear association between postprocedural hsCRP and the risk of death in ACS patients undergoing PCI.

Inflammation after ACS is a delicate balance between healing and damaging, as pro- and anti-inflammatory cells and agents act cohesively to wipe out the necrotic debris and initiate myocardial recovery [12, 16, 17]. As a sensitive serum marker, hsCRP is widely used to measure the intensity of systemic inflammation [1, 2, 18]. However, robust evidence from many studies show that higher perioperative hsCRP is constantly associated with worse outcomes among MI patients treated by PCI [3, 6]. In this study, we showed that both extremely high or low levels of postprocedural hsCRP after PCI for ACS patients were associated with increased mortality. To our knowledge, this is the first study to show a nonlinear association between inflammatory markers and mortality after ACS, which also offered clinical evidence that inflammation imposes dual implications for outcomes.

The current study agrees with previous findings that ACS patients with elevated hsCRP have worse outcomes [3]. A systematic analysis with 6993 patients undergoing PCI for STEMI shows a 2.47-fold increase in the risk of death among patients with elevated periprocedural hsCRP [6]. The reported increase of hazard is similar to the current results, with a 2.02-fold increase for risk of all-cause death among patients in the highest quintile of postprocedural hsCRP. In sum, our results were in line with previous evidence that excessive inflammation after ACS was detrimental for long-term outcomes.

The difference mainly lies in the patients with low level of postprocedural hsCRP. Patients in the lowest quintile of hsCRP presented risk of death as high as those in the highest quintile. The nonlinear association between hsCRP and HR for all-cause and cardiac death was further confirmed by RCS analysis. Although the interpretation could be challenging, the main issue boiled down to the dynamics of hsCRP after ACS and the pathophysiological process behind. As a surrogate marker, hsCRP mainly reflects the activation of the IL-1*β*/IL-6/CRP pathway [19]. IL-6 is the key promoter for CRP synthesis and leucocyte recruitment after myocardial damage [18, 19]. It is released from cardiomyocytes and regulates infiltration of neutrophils and macrophages into infarcted myocardium [16, 20, 21], but failure to gather these cells leads to delayed cleaning of apoptotic or necrotic cardiomyocytes, increased myocardial fibrosis, and reduced ejection fraction [7, 20]. On the other hand, continuous inhibition of IL-6 receptors results in worse cardiac function and increased infarct size [20, 22]. The current study contributed to previous findings by clinically showing the linkage between worsening outcomes and inadequate downstream systemic inflammatory response, as indicated by low hsCRP after ACS. In this study, patients in the lowest quintile presented a median postprocedural hsCRP of only 1.39 (0.95-1.82) mg/L. This suggested a general lack of the upstream activation of the proinflammatory pathway and response for myocardial damage, since the hsCRP level did not increase even after adequate delay before blood tests and ongoing inflammatory stimulus coming from myocardial necrosis, PCI procedures, and reperfusion. As the activation of the proinflammatory pathway is necessary to sustain efficient myocardial healing [23], the increased mortality could be due to delayed recovery caused by lack of acute systemic inflammation. Interestingly, current results showed that low hsCRP was associated with higher all-cause and cardiac mortality, but not the risk of noncardiac death. Also, nonlinearity was not observed for hsCRP and noncardiac death. These findings suggested that the increased mortality associated with low hsCRP might primarily result from the pathophysiological changes within cardiac structures, while the moderate inflammation indicated by intermediate elevation of postprocedural hsCRP (2.26-4.85 mg/L) could be protective and necessary for myocardial recovery. Particularly, the dual effects of inflammation seem to be more pronounced in the subgroup of STEMI, although no interactions were observed between types of ACS and level of hsCRP.

Another issue to be clarified was why a nonlinear association was not observed in previous studies. The dynamics of CRP synthesis makes it necessary to consider the impacts of timing for hsCRP measurements. With a half-life over 19 hours, plasma concentration of CRP is mainly determined by its rate of synthesis, which begins in the liver about 6 hours after the initial inflammatory stimulation [2, 18, 24]. In this study, hsCRP was measured on the next morning after PCI procedure with a median delay of 16.08 hours, which allowed enough time for CRP synthesis in reaction to myocardial necrosis and further damage caused by PCI or reperfusion. Moreover, the level of hsCRP continues to increase following reperfusion and remained stably high for a few days, which is closely related to infarct size, microvascular occlusion, and left ventricular function [11]. Since the reported time to reperfusion is relatively short in most previous studies, periprocedural hsCRP is more associated with plaque vulnerability and original inflammatory status [2, 3]. Thus, the prognostic information offered by peri- and postprocedural hsCRP was different. While the periprocedural hsCRP could help to decide treating strategies for culprit lesions, the postprocedural hsCRP might be more essential for guiding anti-inflammatory medication during the acute phase and long-term risk stratification.

The current study is of clinical importance, as it pointed out a high-risk group of ACS patients with low hsCRP after PCI, who presented risk of death as high as those with significantly elevated hsCRP and required extra attention from physicians. Future research is warranted regarding the etiologies, risk factors, and management for such ACS patients without systemic inflammation.

### 4.1. Limitations

The general limitations of this study are as follows. Firstly, the study was based on a retrospective cohort. Although hsCRP was measured prospectively, the postprocedural blood sample collection could not be precisely specified to the same point of time due to the retrospective design. Residual confounding could exist, and causality between acute inflammation and outcomes could not be fully defined. In addition, this observational study was accomplished in a single large-volume PCI center. The extrapolation of current results still required further validation, while prospective multicenter studies with scheduled hsCRP measurements at different timing are warranted.

## 5. Conclusions

Both low and high postprocedural hsCRP were associated with increased risk of death in patients with ACS treated by PCI.

## Figures and Tables

**Table 1:** Baseline characteristics of study patients by quintiles of postprocedural hsCRP.

Quintile	Overall (*N* = 3940)	Q1 (*N* = 787)	Q2 (*N* = 789)	Q3 (*N* = 788)	Q4 (*N* = 787)	Q5 (*N* = 789)	*P* value
Age (years)	59.0 ± 11.9	58.5 ± 11.3	58.2 ± 11.9	59.2 ± 12.2	58.8 ± 12.0	60.4 ± 12.0	0.002
Male sex, *n* (%)	3105 (78.8)	649 (82.5)	622 (78.8)	626 (79.4)	602 (76.5)	606 (76.8)	0.028
Diabetes mellitus, *n* (%)	1291 (32.8)	241 (30.6)	241 (30.5)	284 (36.0)	254 (32.3)	271 (34.4)	0.084
Hypertension, *n* (%)	2402 (61.0)	443 (56.3)	488 (61.9)	502 (63.7)	484 (61.5)	485 (61.5)	0.038
Killip classes II-IV, *n* (%)	559 (14.2)	63 (8.0)	77 (9.8)	90 (11.4)	138 (17.5)	191 (24.2)	<0.001
STEMI, *n* (%)	3448 (87.5)	684 (86.9)	666 (84.4)	684 (86.8)	706 (89.7)	708 (89.7)	0.006

Previous revascularization							
CABG, *n* (%)	45 (1.1)	13 (1.7)	11 (1.4)	10 (1.3)	5 (0.6)	6 (0.8)	0.270
PCI, *n* (%)	543 (13.8)	132 (16.8)	135 (17.1)	104 (13.2)	89 (11.3)	83 (10.5)	<0.001

Laboratory tests							
LDL-C (mmol/L)	2.7 ± 0.9	2.7 ± 0.9	2.8 ± 0.9	2.8 ± 1.0	2.8 ± 0.9	2.7 ± 0.9	0.001
hsCRP (mg/L)	7.24 (2.77-12.06)	1.39 (0.95-1.82)	3.32 (2.76-3.99)	7.24 (5.97-8.82)	11.60 (11.06-12.05)	13.68 (13.02-14.46)	<0.001
Creatinine (*μ*moI/L)	81.7 ± 23.9	79.6 ± 19.9	79.5 ± 20.5	81.9 ± 23.9	82.6 ± 26.0	85.0 ± 27.9	<0.001

Intraprocedure details							
Culprit lesion, *n* (%)							
LM	96 (2.4)	20 (2.5)	12 (1.5)	16 (2.0)	27 (3.4)	21 (2.7)	0.070
LAD	1734 (44.0)	353 (44.9)	317 (40.2)	338 (42.9)	361 (45.9)	365 (46.3)	
LCX	598 (15.2)	115 (14.6)	124 (15.7)	122 (15.5)	108 (13.7)	129 (16.4)	
RCA	1494 (37.9)	292 (37.1)	332 (42.1)	309 (39.2)	289 (36.7)	272 (34.5)	
Bypass graft	18 (0.5)	7 (0.9)	4 (0.5)	3 (0.4)	2 (0.3)	2 (0.3)	
Multivessel disease, *n* (%)							
1-vessel disease	996 (25.3)	216 (27.5)	196 (24.8)	200 (25.4)	196 (24.9)	188 (23.8)	0.679
2-vessel disease	1249 (31.7)	254 (32.3)	254 (32.2)	250 (31.7)	235 (29.9)	256 (32.5)	
3-vessel disease	1695 (43.0)	317 (40.3)	339 (43.0)	338 (42.9)	356 (45.2)	345 (43.7)	
Pre-PCI TIMI 0 flow, *n* (%)	2598 (65.9)	477 (60.6)	507 (64.3)	502 (63.7)	549 (69.8)	563 (71.4)	<0.001
D2B time (mins)	130 (96-205)	123 (90-202)	122 (94-190)	131 (97-200)	133 (100-216)	138 (101-218)	0.002
Stent placement, *n* (%)	3474 (88.2)	676 (85.9)	710 (90.0)	693 (87.9)	692 (87.9)	703 (89.1)	0.129
Thrombus aspiration, *n* (%)	1648 (41.8)	280 (35.6)	332 (42.1)	327 (41.5)	343 (43.6)	366 (46.4)	<0.001
IABP, *n* (%)	384 (9.8)	56 (7.1)	42 (5.3)	54 (6.9)	105 (13.3)	127 (16.1)	<0.001
Glycoprotein IIb/IIIa inhibitor, *n* (%)	539 (13.7)	89 (11.3)	100 (12.7)	98 (12.4)	129 (16.4)	123 (15.6)	0.012
Post-PCI TIMI 3 flow, *n* (%)	3796 (96.4)	756 (96.1)	768 (97.3)	760 (96.5)	756 (96.1)	756 (95.8)	0.522
Complete revascularization before discharge, *n* (%)	1719 (43.6)	363 (46.1)	355 (45.0)	339 (43.0)	346 (44.0)	316 (40.1)	0.144

Medication							
Aspirin, *n* (%)	3900 (99.0)	782 (99.4)	786 (99.6)	779 (98.9)	777 (98.7)	776 (98.4)	0.089
P2Y12 inhibitors, *n* (%)	3909 (99.2)	784 (99.6)	784 (99.4)	784 (99.5)	778 (98.9)	779 (98.7)	0.178
Statin, *n* (%)	3688 (93.6)	735 (93.4)	730 (92.5)	745 (94.5)	736 (93.5)	742 (94.0)	0.554

CABG: coronary artery bypass grafting; hsCRP: high-sensitivity C-reactive protein; D2B time: door-to-balloon time; IABP: intra-aortic balloon pump; LDL-C: low-density lipoprotein cholesterol; LAD: left anterior descending artery; LCX: left circumflex; LM: left main; PCI: percutaneous coronary intervention; RCA: right coronary artery; STEMI: ST-segment elevated myocardial infraction; TIMI flow: Thrombolysis in Myocardial Infarction grade flow.

**Table 2:** Baseline characteristics of patients by classifications of acute coronary syndromes.

	Overall (*N* = 3940)	NSTE-ACS (*N* = 492)	STEMI (*N* = 3448)	*P* value
Age (years)	59.0 ± 11.9	61.4 ± 11.3	58.7 ± 11.9	<0.001
Male sex, *n* (%)	3105 (78.8)	360 (73.2)	2745 (79.6)	0.001
Diabetes mellitus, *n* (%)	1291 (32.8)	193 (39.2)	1098 (31.8)	0.001
Hypertension, *n* (%)	2402 (61.0)	346 (70.3)	2056 (59.6)	<0.001
Killip classes II-IV, *n* (%)	559 (14.2)	63 (12.8)	496 (14.4)	0.347

Previous revascularization				
CABG, *n* (%)	45 (1.1)	18 (3.7)	27 (0.8)	<0.001
PCI, *n* (%)	543 (13.8)	127 (25.8)	416 (12.1)	<0.001

Laboratory tests				
LDL-C (mmol/L)	2.7 ± 0.9	2.6 ± 0.9	2.8 ± 0.9	<0.001
hsCRP (mg/L)	7.24 (2.77-12.06)	5.55 (2.52-11.30)	7.55 (2.82-12.16)	<0.001
Creatinine (*μ*moI/L)	81.7 ± 23.9	82.7 ± 22.3	81.6 ± 24.1	0.322

Intraprocedure details				
Culprit lesion, *n* (%)				
LM	96 (2.4)	32 (6.5)	64 (1.9)	<0.001
LAD	1734 (44.0)	173 (35.2)	1561 (45.3)	
LCX	598 (15.2)	143 (29.1)	455 (13.2)	
RCA	1494 (37.9)	138 (28.1)	1356 (39.3)	
Bypass graft	18 (0.5)	6 (1.2)	12 (0.4)	
Multivessel disease, *n* (%)				
1-vessel disease	996 (25.3)	92 (18.8)	904 (26.2)	<0.001
2-vessel disease	1249 (31.7)	135 (27.4)	1114 (32.3)	
3-vessel disease	1695 (43.0)	265 (53.9)	1430 (41.5)	
Pre-PCI TIMI 0 flow, *n* (%)	2598 (65.9)	183 (37.2)	2415 (70.0)	<0.001
D2B time (mins)	130 (96-205)	256 (177-621)	121 (93-178)	<0.001
Stent placement, *n* (%)	3474 (88.2)	403 (81.9)	3071 (89.1)	<0.001
Thrombus aspiration, *n* (%)	1648 (41.8)	99 (20.1)	1549 (44.9)	<0.001
IABP, *n* (%)	384 (9.8)	37 (7.5)	347 (10.1)	0.075
Glycoprotein IIb/IIIa inhibitor, *n* (%)	539 (13.7)	29 (5.9)	510 (14.8)	<0.001
Post-PCI TIMI 3 flow, *n* (%)	3796 (96.4)	468 (95.1)	3328 (96.5)	0.122
Complete revascularization before discharge, *n* (%)	1719 (43.6)	216 (43.9)	1503 (43.6)	0.896

Medication				
Aspirin, *n* (%)	3900 (99.0)	486 (98.8)	3414 (99.0)	0.629
P2Y12 inhibitors, *n* (%)	3909 (99.2)	488 (99.2)	3421 (99.2)	0.944
Statin, *n* (%)	3688 (93.6)	466 (94.7)	3222 (93.5)	0.281

CABG: coronary artery bypass grafting; hsCRP: high-sensitivity C-reactive protein; D2B time: door-to-balloon time; IABP: intra-aortic balloon pump; LDL-C: low-density lipoprotein cholesterol; LAD: left anterior descending artery; LCX: left circumflex; LM: left main; NSTE-ACS: non-ST elevation acute coronary syndromes; PCI: percutaneous coronary intervention; RCA: right coronary artery; STEMI: ST-segment elevation myocardial infraction; TIMI flow: Thrombolysis in Myocardial Infarction grade flow.

**Table 3:** Incidence rate and hazard ratio according to quintiles of postprocedural hsCRP for all-cause death, cardiac death, and noncardiac death. HR: hazard ratio; CI: confidence interval; hsCRP: high-sensitivity C-reactive protein.

hsCRP (mg/L)	Incidence rate (/1000 person-year)	Unadjusted HR (95% CI)	*P* value	Adjusted HR (95% CI)	*P* value
All-cause death					
<2.26	13.70	1.65 (0.94-2.87)	0.079	1.90 (1.08-3.33)	0.025
2.26-4.85	8.39	1 (reference)	—	1 (reference)	—
4.85-10.16	14.06	1.67 (0.97-2.89)	0.066	1.31 (0.75-2.30)	0.337
10.16-12.56	18.67	2.21 (1.31-3.73)	0.003	1.86 (1.09-3.16)	0.023
≥12.56	24.93	2.99 (1.82-4.91)	<0.001	2.02 (1.21-3.36)	0.007

Cardiac death					
<2.26	10.38	2.07 (1.04-4.13)	0.038	2.39 (1.19-4.80)	0.014
2.26-4.85	5.04	1 (reference)	—	1 (reference)	—
4.85-10.16	7.81	1.56 (0.76-3.19)	0.223	1.19 (0.57-2.47)	0.642
10.16-12.56	13.11	2.61 (1.35-5.06)	0.004	1.99 (1.01-3.91)	0.046
≥12.56	16.15	3.32 (1.76-6.27)	<0.001	1.96 (1.01-3.77)	0.045

Noncardiac death					
<2.26	3.32	1.00 (0.38-2.67)	0.999	1.12 (0.41-3.00)	0.828
2.26-4.85	3.36	1 (reference)	—	1 (reference)	—
4.85-10.16	6.25	1.82 (0.78-4.26)	0.166	1.49 (0.63-3.54)	0.368
10.16-12.56	5.56	1.62 (0.68-3.86)	0.278	1.55 (0.65-3.74)	0.326
≥12.56	8.78	2.49 (1.12-5.53)	0.025	2.00 (0.89-4.53)	0.095

**Table 4:** Adjusted hazard ratio according to quintiles of postprocedural hsCRP in the subgroups of STEMI and NSTE-ACS.

	STEMI (*N* = 3448)		NSTE-ACS (*N* = 492)		
hsCRP (mg/L)	Adjusted HR (95% CI)	*P* value	Adjusted HR (95% CI)	*P* value	*P*_interaction_
All-cause death					
<2.26	1.84 (0.98-3.45)	0.057	5.84 (1.18-28.79)	0.030	0.708
2.26-4.85	1 (reference)	—	1 (reference)	—	
4.85-10.16	1.30 (0.70-2.40)	0.406	2.58 (0.48-14.04)	0.272	
10.16-12.56	1.91 (1.07-3.40)	0.029	2.31 (0.37-14.58)	0.371	
≥12.56	2.15 (1.23-3.76)	0.007	1.53 (0.30-7.92)	0.609	

Cardiac death					
<2.26	2.84 (1.24-6.48)	0.013	3.51 (0.65-19.07)	0.146	0.509
2.26-4.85	1 (reference)	—	1 (reference)	—	
4.85-10.16	1.36 (0.58-3.19)	0.484	1.29 (0.20-8.15)	0.787	
10.16-12.56	2.48 (1.13-5.43)	0.023	0.41 (0.03-5.05)	0.485	
≥12.56	2.44 (1.12-5.28)	0.024	0.99 (0.17-5.63)	0.989	

HR: hazard ratio; CI: confidence interval; hsCRP: high-sensitivity C-reactive protein; NSTE-ACS: non-ST elevation acute coronary syndromes; STEMI: ST-segment elevation myocardial infraction.

## Data Availability

The data used to support the findings of this study are available from the corresponding author upon request.
